# Complete reversal of bilateral optic nerve infiltration from lymphoblastic leukemia using chemotherapy without adjuvant radiotherapy

**DOI:** 10.1186/s12886-021-02097-w

**Published:** 2021-09-15

**Authors:** Douglas Rodrigues da Costa, Rodrigo Dahia Fernandes, Fernanda Nicolela Susanna, Epitácio Dias da Silva Neto, Mario Luiz Ribeiro Monteiro

**Affiliations:** Faculdade de Medicina FMUSP, Universidade de São Paulo, São Paulo, SP Brazil

**Keywords:** Leukemic infiltration, Acute lymphoblastic leukemia

## Abstract

**Background:**

Leukemic involvement of the eyes is rare, therefore, treatment relies on previous case reports. The treatment of ocular complications poses additional difficulties, because the eye is considered as a pharmacological “sanctuary” for patients with acute lymphoblastic leukemia (ALL). Therefore, radiotherapy is the main therapeutic choice; however, it might lead to many important side effects. To the best of our knowledge, this is the first case report of a bilateral leukemic optic nerve infiltration that remitted with chemotherapy without adjuvant radiotherapy.

**Case presentation:**

A 30-year-old female patient with previous history of remitted ALL presented with a one-week history of floaters in her right eye. Her ophthalmological exam showed remarkable optic disc swelling, in both eyes. She was diagnosed with ALL relapse presenting as a bilateral optic nerve leukemic infiltration. Local radiotherapy was planned for both eyes, however, due to efficient recovery with chemotherapy, it was cancelled. Allogenic bone marrow transplantation was subsequently performed. The patient is being followed up and ALL remitted.

**Conclusion:**

Leukemia relapse on central nervous system, despite rare, is a sign of poor prognosis and requires prompt treatment. Its occurrence on ocular tissues is even rarer. It is hypothesized that the blood-brain barrier limits the delivery of chemotherapeutic drugs to the eye and infiltration of the optic nerve by leukemic cells might prejudice the flow of cerebrospinal fluid between the cranial space and the optic disc.

**Supplementary Information:**

The online version contains supplementary material available at 10.1186/s12886-021-02097-w.

## Background

Leukemic involvement of the eyes and central nervous system (CNS) is becoming more frequent as the patients’ survival rate increases. Despite efficient combined therapy, recurrence of the disease is both a diagnostic and therapeutic challenge, especially when it occurs in unusual sites, as the eye [1]. The treatment of ocular complications poses additional difficulties, because the eye is considered as a pharmacological “sanctuary” for patients with acute lymphoblastic leukemia (ALL) [2]. Given the rarity of its occurrence, the chosen therapeutic method is local radiotherapy and is based on previous case reports [3–5]. However, local radiotherapy might lead to many ophthalmologic complications, such as radiation-induced dry eye, scleral necrosis, cataract, retinopathy and optic neuropathy [6]. This case report presents a relapse of ALL manifesting as bilateral optic nerve infiltration that responded to chemotherapy without adjuvant radiotherapy.

## Case presentation

In August 2020, a 30-year-old female patient presented with a one-week history of floaters in the right eye (OD). She had past medical history of B-cell acute lymphoblastic leukemia with chromosomal translocation t(1;19)(q23; p13.3) TCF3/PBX1 diagnosed in July 2018. Her treatment began in August 2018, with 6-mercaptopurine, vincristine, methotrexate, prednisone, cytarabine, idarubicin, prophylactic intrathecal methotrexate and dexamethasone. Systemic remission was achieved in May 2020. Before hospital discharge, neurological and ophthalmological exams were normal.

**Fig. 1:**
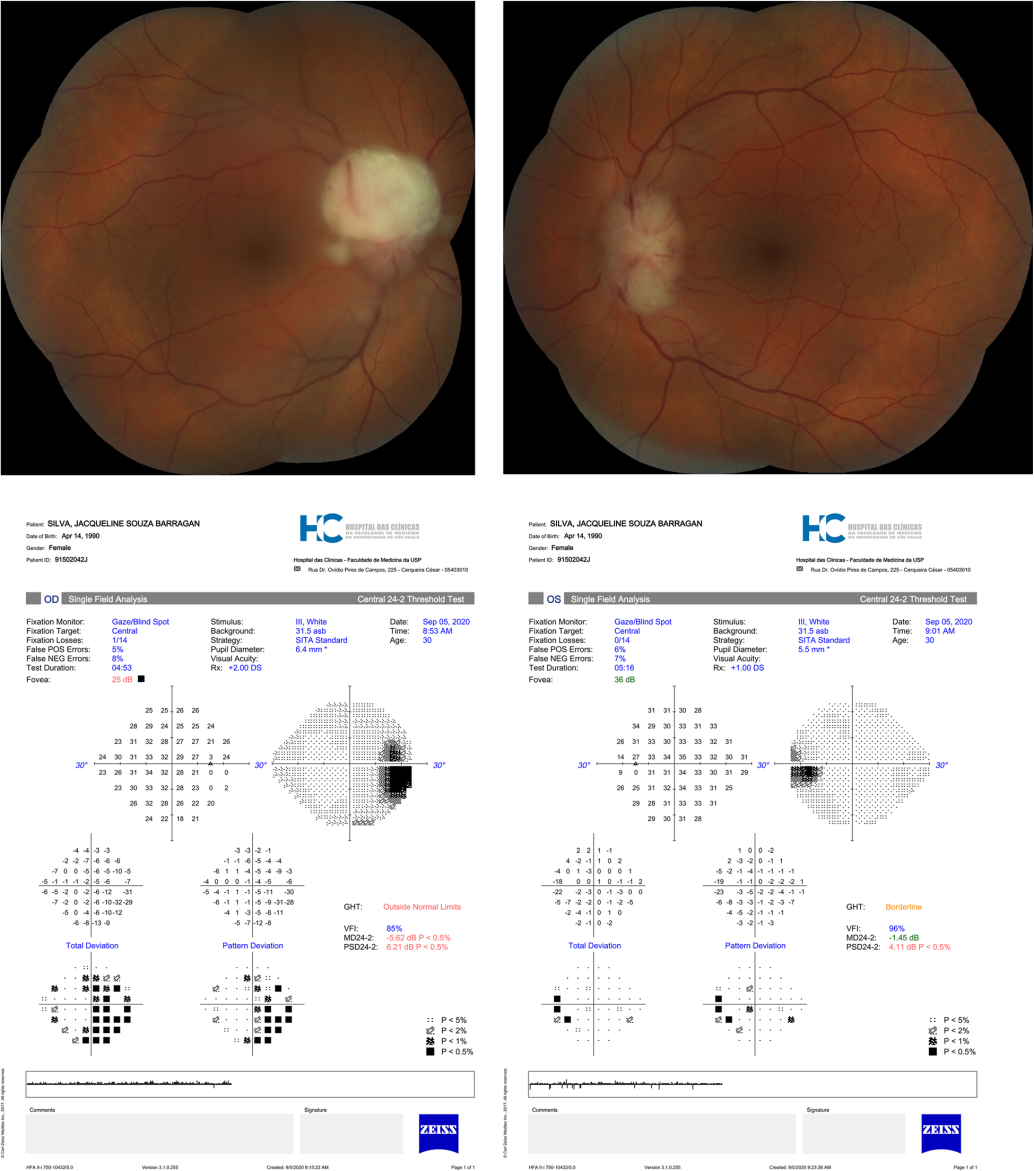
Fundus photos of both eyes and corresponding visual fields (Central 24–2 Threshold Test, SITA Standard), both acquired at the same day, before chemotherapy was initialized. Visual field examinations were reliable. (**A**) Fundus photo of the right eye presents optic disc edema and white “creamy” mass, folding towards the macular region. Above, visual field with marked enlargement of the blind spot. **(B)** Fundus photo of the left eye with optic disc edema, less pronounced than OD. Visual field had a minor blind spot enlargement

**Fig. 2:**
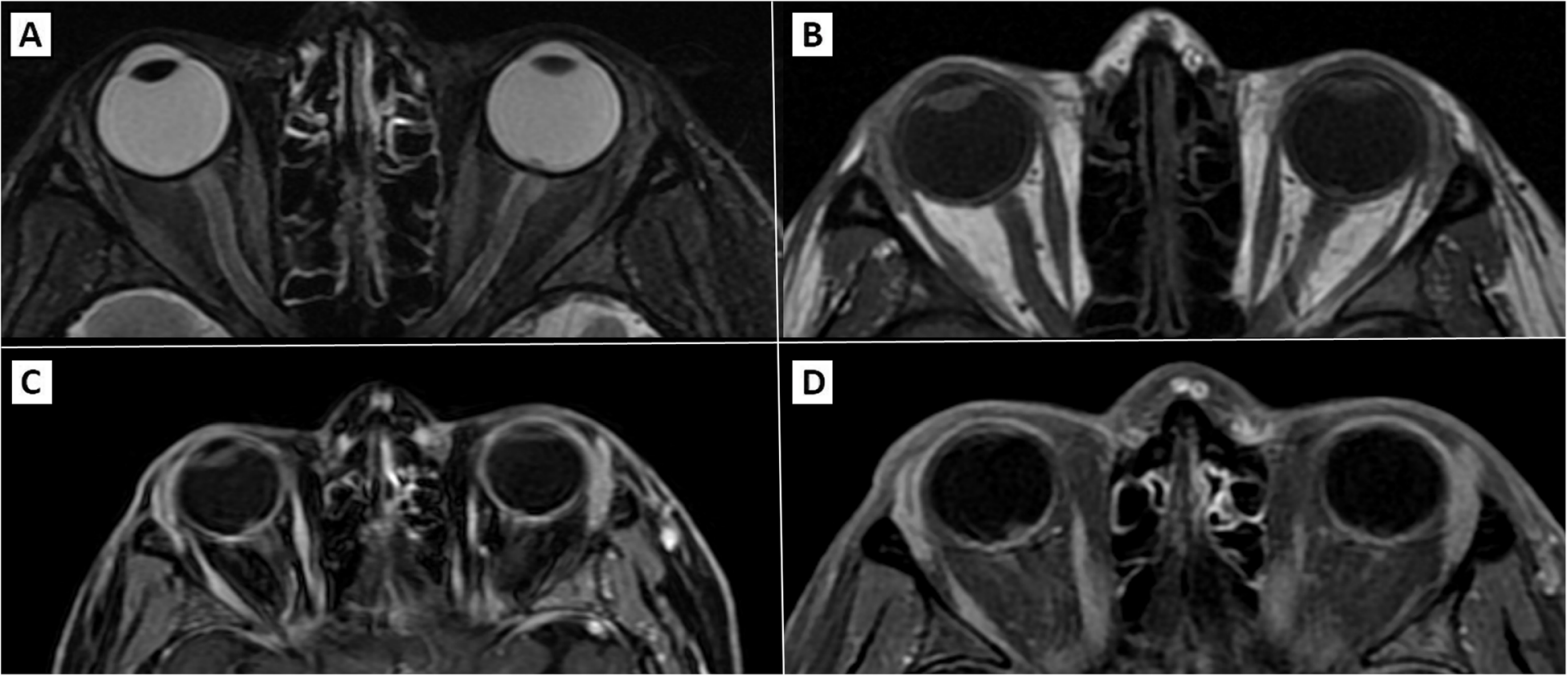
Left and right orbits MRI scans with presence of small nodules on both optic disks, especially on the right eye, which was compatible with bilateral leukemic infiltration. Optic nerves, chiasma, and other neurological segments were unaffected. **(A)**: T2-weighted, a small nodule is best evidenced on the left eye; **(B)**: T1-weighted without contrast and fat suppression, evidencing no impairment of optic nerves; C/D: T2-weighted with gadolinium contrast, small nodules of the left eye are highlighted by the gadolinium contrast

Due to her visual complaints, she was referred for an ophthalmological examination. Visual acuity was 20/20 OU, biomicroscopic examination and pupillary reflexes were unremarkable. On ophthalmoscopy, there was peripapillary nerve fiber layer edema with protrusion of a white mass with a “creamy” aspect from the optic disc on both eyes (OU). There were small traces of white “creamy” material on inferior vitreous on OD. Macula was normal OU. Visual field showed enlargement of the blind spot in OD and was normal in the left eye (OS) (Fig. 1). Orbit and head magnetic resonance imaging (MRI) scan showed small low intensity nodules on optic nerve head OU, which were classified as compatible with ALL infiltration (Fig. 2). Neurological examination was normal. Lumbar puncture (LP) showed normal cerebrospinal fluid (CSF) open pressure (17 cm), neoplastic cells and positive immunofluorescence markers for leukemia. There were no other sites of CNS affection. The final diagnosis was of bilateral optic nerve leukemic infiltration as initial site for disease relapse.

**Fig. 3:**
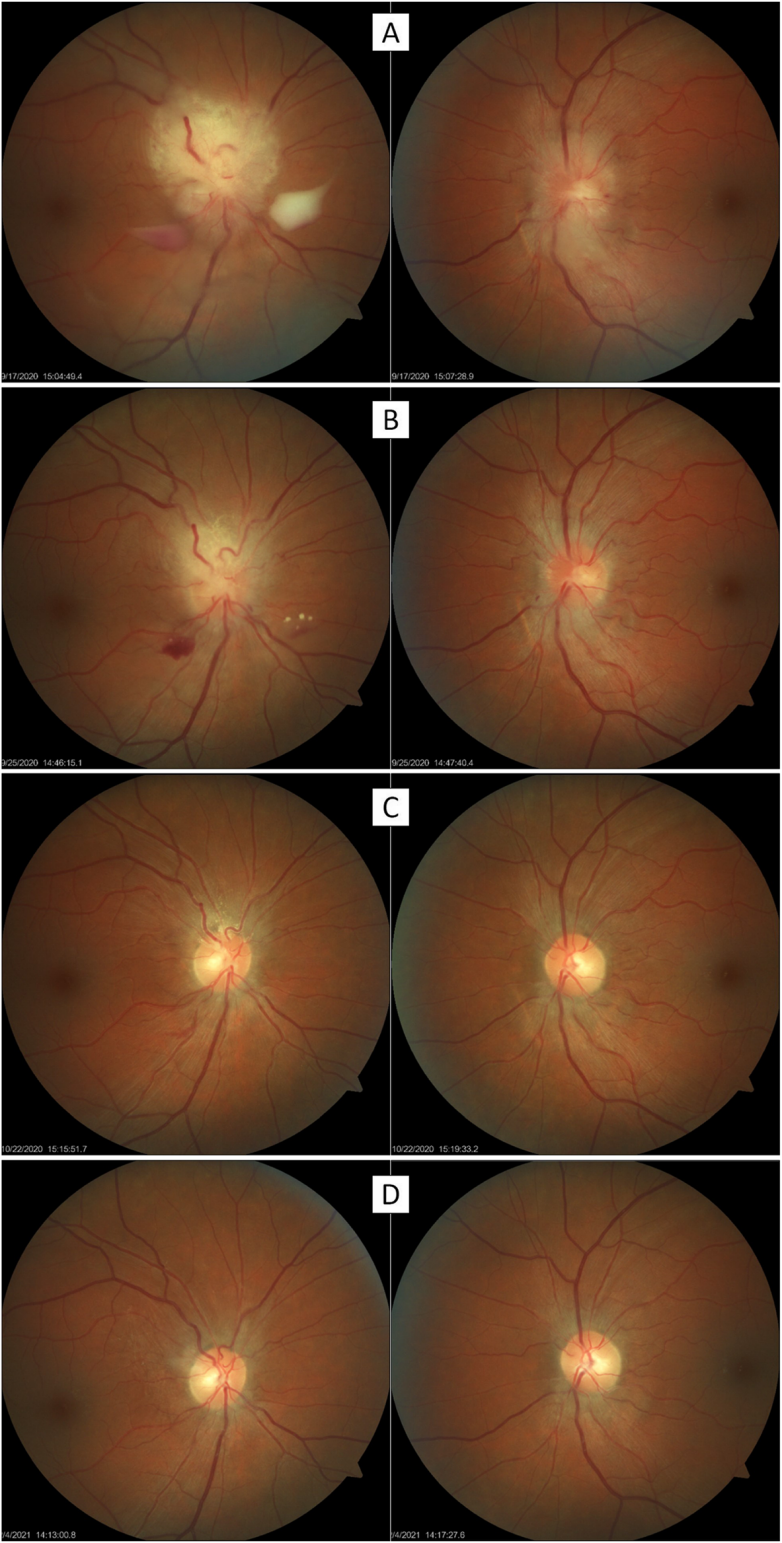
Optic nerve head during treatment. OD and OS are on the left and right columns, respectively. **(A)** Three days after the first chemotherapy dose. Remarkable shrinkage of the white infiltrative mass was noticed. On OD, there were some whitish remnants on the vitreous inferiorly. **(B)** Ten days of treatment. Vitreous was clear OU and OD showed prominent peripapillary vascular tortuosity and a small pre-retinal hemorrhage, without signs of vascular occlusion. In the OS the superior and inferior disc margins were slightly blurry. **(C)** Twenty-one days of treatment. OD vascular tortuosity was less pronounced, and the hemorrhage had been absorbed. OS examination was unremarkable. **(D)** 60 days after allogenic transplantation. There was minor optic pallor OD temporally and vitreous was normal. OS remained unchanged

Bone marrow transplant was programmed after full hyper-CAVD drug combination chemotherapy (cyclophosphamide, vincristine sulfate, doxorubicin hydrochloride and dexamethasone) and intrathecal methotrexate cycle, which began in August 2020. Local radiotherapy OU was planned but on the third day after starting chemotherapy (D3), ophthalmoscopy showed remarkable improvement OU (Fig. 3A). Therefore, radiotherapy was postponed). On D10 the patient had no ophthalmological complaints, no visual field defects, clear vitreous OD and marked improvement of the disc appearance OU (Fig. 3B). Local radiotherapy planning was therefore cancelled. On D21, ophthalmoscopy revealed small reminiscences of optic disc leukemic infiltration on OD (Fig. 3C). Immunosuppressive therapy was initiated for bone marrow transplant and no further photographical registration could be done until hospital discharge.

In December 2020, the patient was submitted to an allogenic bone marrow transplant. In February 2021 she had no ophthalmic complaints, and her exams were all normal. On ophthalmoscopy, OD showed minor optic disc pallor and OS was normal (Fig. 3D). Acute lymphoblastic leukemia remitted, and the patient is being followed up closely until the date. Upon any changes in the patient’s status, prompt necessary treatment will be initiated.

## Discussion and conclusion

Leukemia relapse on CNS, despite rare, is a sign of poor prognosis and requires prompt treatment. Its occurrence on ocular tissues is even rarer. Hence, management is mainly based on case reports and pharmacological theories over the chemotherapeutic agents’ mechanisms on the eye. Previous studies reported partial or unsuccessful attempts of systemic and intrathecal chemotherapy for optic nerve involvement [3]. Adjunct local radiotherapy has been the chosen modality [1, 3, 6–11].

It is hypothesized that the blood-brain limits the delivery of chemotherapeutic drugs to the eye [4] and infiltration of the optic nerve by leukemic cells might prejudice the flow of CSF between the cranial space and the optic disc. As these cells are highly sensitive to radiation, radiotherapy is the natural choice. However, it has important side-effects as radiation-induced optic neuropathy, cataract, dry-eye, secondary glaucoma from iris neovascularization, retinopathy, and increased risk of leukoencephalopathy [5, 6, 12].

Leukemia can lead to ophthalmologic complications not only through direct infiltration [13]. Its pathophysiology can be majorly divided either in primary complications due to tumor cells infiltration, or in secondary complications by hematological abnormalities such as thrombocytopenia, hyperviscosity, errythrocytopenia, and opportunistic infections [13]. Immediate comprehensive examination must be tackled to diagnose the underlying pathophysiology and to exclude other conditions, mainly optic neuritis, papilledema, and other causes of optic nerve disc swelling, including ischemic optic neuropathy, compressive optic neuropathy and ischemic optic neuropathy [13]. Regarding primary complications, the infiltration posteriorly to the lamina cribosa is related to more serious symptoms, such as central retinal artery occlusion, central retina vein occlusion, neovascular glaucoma and retinal detachment, whilst the impairment of its anterior portion might even be asymptomatic [14].

Previous studies mainly reported radiotherapy use for optic nerve infiltration by leukemic cells. As this disease mainly affects children, and also because they have better survival and remission rates when compared to non-infants, most case reports refer to the pediatric population [7]. Within that group, good outcomes upon adjunct local radiation for unilateral infiltration, and cerebral radiation for bilateral cases have been reported by authors [1, 8–11].

Although the remission and survival rates for the adult population have significantly improved, case reports on ophthalmologic impairments are still rare. And, to our knowledge, in all reported cases, which included chronic and acute leukemia, patients were managed with adjunct radiotherapy [3, 5, 15, 16]. Regarding ALL patients, a total of 5 were reported, all treated with adjunct radiotherapy: in two patients vision recovered without further complications [3, 15]; in one case there was improvement of leukemic infiltration, however complete visual recovery was not achieved as the optic nerve developed optic atrophy due to radiation [8]; two patients recovered from infiltration but unfortunately did not survive through the whole treatment [3, 7]. Finally, there is a single patient, who recovered fully with chemotherapy solely, however, it was an asymptomatic pediatric patient, with unilateral infiltration [14].

To the best of our knowledge, this is the first case report of a bilateral optic nerve infiltration in ALL that responded to chemotherapy without adjunct radiotherapy. In our patient, intrathecal methotrexate was initiated by the oncology team and due to the fast response local radiotherapy planning was cancelled. The main limitation of this article is that it is a single case report and, therefore, our findings cannot be generalized for further treatment of similar cases. Hence, this case addresses the action of chemotherapeutic drugs on ocular tissues and reinforces the importance of ophthalmologic examination during and after treatment of leukemia. We do not intend to suggest the sole use of systemic chemotherapy for leukemic infiltration of the optic nerve, but rather encourage further studies on chemotherapeutic drugs and possibly avoid unnecessary radiation of the eye.

## Supplementary Information

**Additional file 1.** Bone marrow analysis upon diagnosis. Bone marrow analysis upon diagnosis. CD: cluster of differentiation; TdT: terminal deoxynucleotidyl transferase; PBX1: PBX Homebox 1.

## Data Availability

All photos and patient’s data are available on physical folders.

## Abbreviations

ALL: Acute lymphoblastic leukemia
OD: Oculus dextrus (right eye)
OS: Oculus sinistrus (left eye)
OU: Oculus uterque (both eyes)
CNS: Central nervous system
CSF: Cerebrospinal fluid
