# Supplementary material for: Complete reversal of bilateral optic nerve infiltration from lymphoblastic leukemia using chemotherapy without adjuvant radiotherapy
Source: BMC Ophthalmol. 2021 Sep 15;21:335. doi: 10.1186/s12886-021-02097-w (PMC8444571; doi:10.1186/s12886-021-02097-w)
Supplement: Supplementary file 1 — Additional file 1. Bone marrow analysis upon diagnosis. Bone marrow analysis upon diagnosis. CD: cluster of differentiation; TdT: terminal deoxynucleotidyl transferase; PBX1: PBX Homebox 1. [file 12886_2021_2097_MOESM1_ESM.pdf]

**Mielogram evaluation**	
	96,4% of blasts; small size cells; hemodiluted and highly hypocellular
**Immunofluorescence markers**	
	Positive B-antigens: CD10, CD19, CD22, citoplasmatic CD79a, citoplasmátic IgM, CD38, HLA-DR, TdT, CD58<br>Negative for CD3, CD4, CD7, CD8, CD13, CD20, CD33, CD34, CD117 and mieloperoxidase
**Biomolecular study**	
	BCR-ABL p190 and p210: negative<br>PBX1: positive in the first phase

Bone marrow analysis upon diagnosis. CD: cluster of differentiation; TdT: terminal deoxynucleotidyl transferase; PBX1: PBX Homeobox 1
